# Virus-Like Particles of SARS-Like Coronavirus Formed by Membrane Proteins from Different Origins Demonstrate Stimulating Activity in Human Dendritic Cells

**DOI:** 10.1371/journal.pone.0002685

**Published:** 2008-07-16

**Authors:** Bingke Bai, Qinxue Hu, Hui Hu, Peng Zhou, Zhengli Shi, Jin Meng, Baojing Lu, Yi Huang, Panyong Mao, Hanzhong Wang

**Affiliations:** 1 State Key Laboratory of Virology, Wuhan Institute of Virology, Chinese Academy of Sciences, Wuhan, People's Republic of China; 2 Graduate School of the Chinese Academy of Sciences, Beijing, People's Republic of China; 3 Department of Virology, Institute of Infectious Disease, Beijing, People's Republic of China; 4 Chinese People's Liberation Army Postgraduate Medical School, Beijing, People's Republic of China; AIDS Research Center, Chinese Academy of Medical Sciences and Peking Union Medical College, China

## Abstract

The pathogenesis of SARS coronavirus (CoV) remains poorly understood. In the current study, two recombinant baculovirus were generated to express the spike (S) protein of SARS-like coronavirus (SL-CoV) isolated from bats (vAcBS) and the envelope (E) and membrane (M) proteins of SARS-CoV, respectively. Co-infection of insect cells with these two recombinant baculoviruses led to self-assembly of virus-like particles (BVLPs) as demonstrated by electron microscopy. Incorporation of S protein of vAcBS (BS) into VLPs was confirmed by western blot and immunogold labeling. Such BVLPs up-regulated the level of CD40, CD80, CD86, CD83, and enhanced the secretion of IL-6, IL-10 and TNF-α in immature dendritic cells (DCs). Immune responses were compared in immature DCs inoculated with BVLPs or with VLPs formed by S, E and M proteins of human SARS-CoV. BVLPs showed a stronger ability to stimulate DCs in terms of cytokine induction as evidenced by 2 to 6 fold higher production of IL-6 and TNF-α. Further study indicated that IFN-γ+ and IL-4+ populations in CD4+ T cells increased upon co-cultivation with DCs pre-exposed with BVLPs or SARS-CoV VLPs. The observed difference in DC-stimulating activity between BVLPs and SARS CoV VLPs was very likely due to the S protein. In agreement, SL-CoV S DNA vaccine evoked a more vigorous antibody response and a stronger T cell response than SARS-CoV S DNA in mice. Our data have demonstrated for the first time that SL-CoV VLPs formed by membrane proteins of different origins, one from SL-CoV isolated from bats (BS) and the other two from human SARS-CoV (E and M), activated immature DCs and enhanced the expression of co-stimulatory molecules and the secretion of cytokines. Finding in this study may provide important information for vaccine development as well as for understanding the pathogenesis of SARS-like CoV.

## Introduction

Severe acute respiratory syndrome (SARS) is an emerging infectious disease caused by a novel coronavirus (CoV) variant, SARS CoV [1]–[5]. During the 2002–2003 epidemic, SARS CoV was highly transmissible in humans. As of 31 December 2003, 8,096 cases had been identified worldwide and 774 people had died with a mortality rate of about 9.6% (World Health Organization statistics (http://www.who.int/csr/sars/country/table 2004_04_21/en/). Although the SARS epidemic was successfully contained by July 2003, the pathogenesis of SARS CoV remains poorly understood.

The viral envelope of SARS CoV contains at least three structural membrane proteins, spike (S), membrane (M), and small membrane or envelope (E) proteins [6], [7]. The cellular receptor of SARS CoV and the receptor-binding domain (RBD) on S protein have been identified, indicating that RBD in the S1 region of S protein plays a critical role in neutralizing antibody induction, angiotensin-converting enzyme 2 (ACE-2) binding and viral entry [8]–[10]. In the absence of S protein, expression of M and E proteins does not induce a detectable serum SARS-CoV-neutralizing antibody response [11]. M and E proteins are the only requirements for the assembly of viral particles; the S protein is dispensable but is incorporated when present [12]–[16].

The isolation of SARS CoV from Himalayan palm civets (*Paguma larvata*) and raccoon dogs (*Nyctereutes procyonoides*) in wild live markets in China suggests that the virus may be originally from animal reservoirs [17]. Recently, two independent groups reported the identification of SARS-like coronavirus (SL-CoV) in different Chinese horseshoe bat species, providing strong evidence that bats could serve as natural reservoirs of SARS CoV [18]–[20]. The S coding sequence of SL-CoV isolated from bats displays low level of similarity to those isolated from human (76%) or civets (78%) [18]–[20], while the E and M proteins of SL-CoV have high sequence similarity (96% to 100%) to those from human or civets [19], [20]. It is worthy to note that the nucleotide sequence identity among these coronaviruses from different sources was greatly increased when the S region was excluded [19], [20]. The host range of coronaviruses was reported to correlate with the degree of S protein variations [21], suggesting that the variations in S protein are responsible for the interspecies transmission and the adaptation to new hosts.

Virus-like particles (VLPs) represent a specific class of subunit vaccine that mimics the structure of authentic virus particles. VLPs present viral antigens in a more authentic conformation than other subunit vaccines and are readily recognized by the immune system [22]. Many studies have documented that VLPs are capable of inducing B-cell-mediated immune responses and are highly effective in stimulating CD4 T cell proliferation and cytotoxic T lymphocyte (CTL) responses [23]–[25]. The stimulating activities of Ebola, Marburg and HIV VLPs in dendritic cells (DCs) were previous investigated [26]–[28], indicating that, unlike native virus, VLPs are effective stimulators of DCs and can enhance innate and adaptive immune responses. Our previous study described that VLPs formed by E, M and S proteins of SARS CoV elicited strong SARS CoV-specific humoral and cellular immune responses in mice [29]. However, whether SL-CoV VLPs can elicit cytokines secretion or/and activate human DCs remains to be determined.

In this study, we have investigated whether the S protein of SL-CoV isolated from bats can incorporate into VLPs formed by the E and M proteins of human SARS CoV. In addition, because in vitro infection model of bat SL-CoV has not so far been established, we intended to use VLPs as an alternative to study the immune responses induced in DCs. Therefore, we compared the phenotypic and functional changes of immature DCs inoculated with BVLPs or with SARS CoV VLPs. The S-specific immune activation was further confirmed in mice using S DNA vaccines. Findings described in this report may have significance for understanding the evolution and the pathogenesis of SARS CoV.

## Results

### Construction of BVLPs

The *bs* gene was amplified by RT-PCR and cloned into pFastBac DUAL vector. The recombinant baculovirus vAcBS was generated following transfection in sf21 cells. The recombinant baculovirus vAcME, expressing the E and M proteins of SARS CoV WH20 strain (GenBank accession number AY772062), was previously described [29].

**Figure 1:**
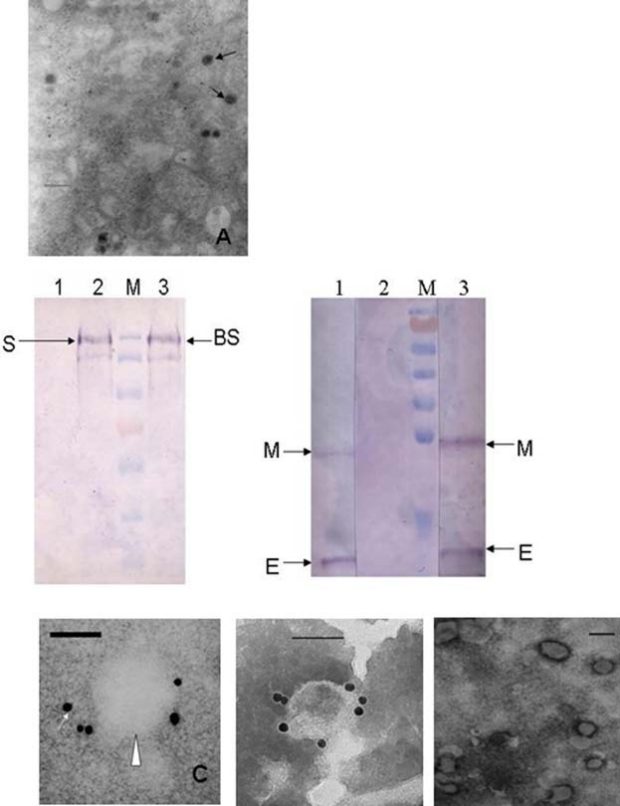
Confirmation of the incorporation of BS protein in BVLPs. (A) Budding of BVLPs from insect cells. Sf21 cells were co-infected with recombinant baculovirus vAcBS and vAcME at a moi of 5. At 72 h post-infection, cells were harvested and fixed with 2% glutaraldehyde and then with 1% osmium tetroxide. Thin-section samples were prepared and stained with 1% uranyl acetate followed by examination under a transmission electron microscope. Arrows indicates BVLPs. Bar = 250 nm. (B) Western blot analysis of BVLPs. 5 µg purified BVLPs were analyzed by western blot using rabbit-derived anti-SARS-CoV antibody. An equivalent amount of purified human SARS CoV VLPs was run in parallel. Preimmune rabbit antiserum was used as negative control. Left part, band corresponds to S protein. Lane 1, negative control; lane 2, human SARS CoV VLPs; lane 3, BVLPs. Right part, band corresponds to E or M protein. Lane 1, human SARS CoV VLPs; lane 2, negative control; lane 3, BVLPs. The protein marker was Fermentas #SM0671. (C) Detection of BVLPs by immunogold labeling. The collodion-coated EM grids were loaded with purified BVLPs (left), SARS CoV VLPs (middle) or VLPs without BS or S protein (right) for 5 min. After removal of the excess sample solution, grids were incubated with antibody specific against BS2 protein for 1 h and then incubated with 15 nm gold conjugated anti-rabbit IgG.. The samples were stained with 2% PTA for 1 min, drained and examined under the EM. Arrow indicates the gold particles and triangle indicates the VLPs. Bar = 100 nm.

VLPs formed by the S, E and M proteins of SARS CoV were successfully constructed in our previous study using a baculovirus system [30]. The main variation between SL-CoV and SARS CoV of human or civet locates in the S protein [18]–[20]. To study whether BS protein can be incorporated into VLPs formed by the E and M proteins of SARS-CoV, we co-infected sf21 cells with the recombinant baculoviruses vAcBS and vAcME. After 72 h of co-infection, the cells were collected and thin-sections were prepared and examined. As shown in Figure 1A, numerous viral particles, ∼100 nm in diameter, were mainly observed in the cytoplasm of infected cells. Western blot and immunogold labeling were subsequently performed to detect the incorporation of BS in VLPs. As shown in Figure 1B, the presence of BS, M and E proteins was detected in BVLPs by using an anti-SARS CoV antibody. There were no corresponding bands in the negative control of preimmune rabit antiserum. The genomic differences between SL-CoV and SARS CoV largely locate in the S gene, particularly at the 5′ end of S region (equivalent to S1 coding region). Therefore, it is not surprising that serum raised against human SARS CoV recognized bat SL-CoV S protein. The S2 coding region of S protein is highly conserved (∼96%) [5], [10], [21]. Using a specific anti-BS2 (Bat S2 region 667–1242 aa) antibody (Figure 1C), the gold particles were detected around the BVLPs in immunogold labeling analysis, further confirming the results of western blot. In addition, the anti-BS2 antibody also recognized the human SARS CoV VLPs but not VLPs formed by E and M proteins in the absence of S protein (Figure 1C). Taken together, our data indicate that the BS protein of SL-CoV was successfully incorporated into VLPs formed by the E and M proteins of human SARS CoV.

### Phenotype characterization of DCs treated with BVLPs

**Figure 2:**
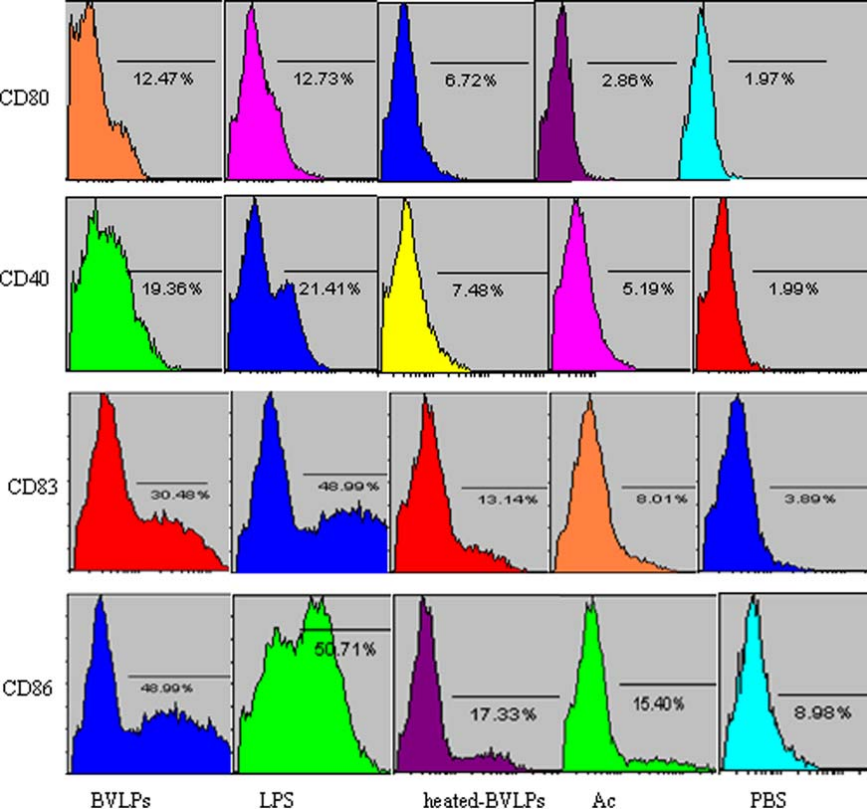
BVLPs induced phenotype changes in DCs. Immature DCs (10^6^cells/ml) were incubated with 10 µg/ml of BVLPs, 10 µg/ml of LPS, 10 µg/ml of heated BVLPs, Ac (culture supernatant of sf21cells transfected with baculovirus expression vector pFastBac DUAL with *gfp* under the control of p10 promoter) or PBS. 16 h post incubation, cells were collected, washed, stained with indicated antibodies, and analyzed by flow cytometry. One representative experiment out of 3 is shown.

Immature DCs were incubated with 10 µg/ml BVLPs for 16 h. The expression of costimulatory molecules (CD40, CD86 and CD80) and the maturation marker CD83 were analyzed. DCs treated with PBS or Ac were used as negative controls. As shown in Fig. 2, similar to those on LPS-treated DCs, all of the four markers were up-regulated to much higher levels than those on PBS or Ac-treated DCs (Fig. 2). Collectively, our results indicate that BVLPs can lead to the activation and maturation of DCs. In contrast, the heated BVLPs induced much lower levels of CD83, CD40, CD86 and CD80 expression on DCs, suggesting that the structural proteins incorporated into VLPs are essential for DC activation and maturation.

### Assays of cytokines in BVLPs stimulated DCs

**Figure 3:**
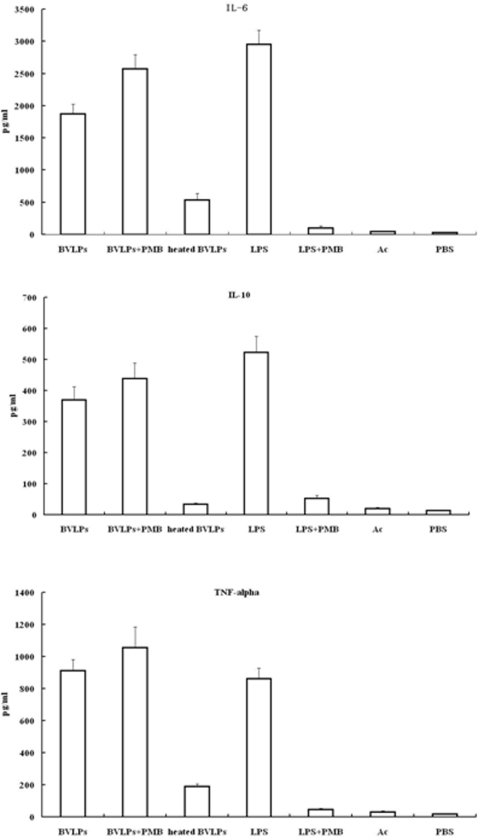
BVLPs induced secretion of cytokines in DCs. DCs were incubated with BVLPs (10 µg/ml), heated BVLPs (10 µg/ml), LPS (10 µg/ml), Ac (culture supernatant of sf21 cells transfected with baculovirus expression vector pFastBac DUAL with *gfp* under the control of p10 promoter), or PBS. BVLPs + PMB or LPS + PMB: 10 µg/ml BVLPs or 10 µg/ml LPS was treated with 10 µg/ml polymyxin B at room temperature for 1 h before incubation with DCs. Following 16 h of treatment, supernatants were collected, and IL-6, IL-10 and TNF-α were analyzed using ELISA. Data are expressed as the mean±SD of triplicate samples.

DC maturation not only leads to up-regulation of adhesion and co-stimulatory molecules, but also results in secretion of proinflammatory cytokines that can activate innate and adaptive immune responses [31]. To assess the effects of BVLPs on DCs, immature DCs were treated under various conditions and supernatants were collected for cytokine (IL-6, IL-10 and TNF-alpha) quantification. IL-6 and TNF-α are proinflammatory cytokines while IL-10 is an anti-inflammatory cytokine. As shown in Fig. 3, incubating DCs with BVLPs significantly enhanced the secretion levels of these three cytokines and were all similar to those in LPS-treated DCs. The boiled-BVLPs lost the ability to induce cytokine production. The secretions of IL-6, IL-10 and TNF-α were 5–10 folds lower in heated BVLPs-treated DCs than those in BVLPs-treated DCs, although the secretion levels were still 5–15 folds higher than those in PBS or Ac treated groups. This enhancement is likely due to non-specific stimulation by remaining denatured proteins in heated BVLPs. Moreover, cytokine secretion profiles were similar in DCs inoculated with BVLPs with or without polymyxin B pretreatment, excluding the possibility of LPS contamination. In contrast, LPS-treated DCs significantly lost the cytokine-secretion ability with polymyxin B pretreatment. Combining the flow cytometry results in Fig. 2, it is reasonable to draw a conclusion that the structure of BVLPs, not LPS contamination, contributed to cytokine production in BVLPs-treated DCs.

### Phenotypic and functional changes of DCs treated with BVLPs or SARS CoV VLPs

**Figure 4:**
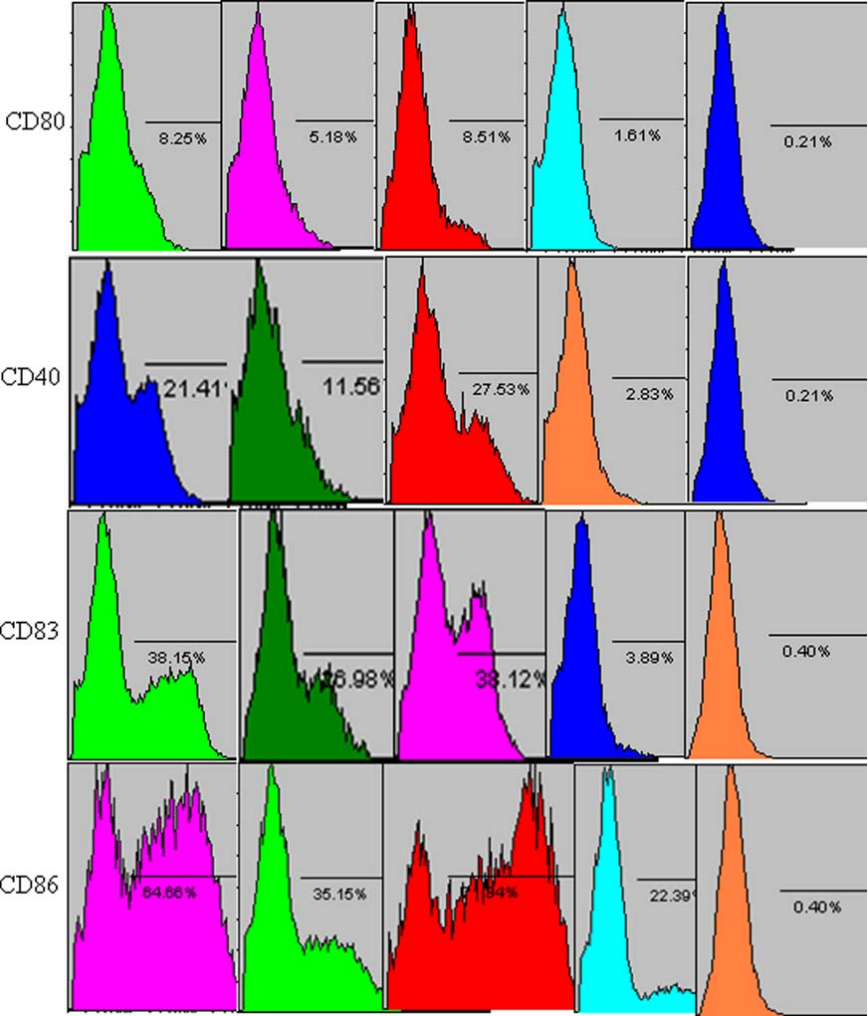
Phenotypic characterization of DCs treated with BVLPs or with SARS CoV VLPs. DCs were incubated with SL-CoV BVLPs or SARS CoV VLPs at a concentration of 10 µg/ml. DCs treated with LPS (10 µg/ml) and PBS were used as positive and negative control, respectively. The cells were harvested at 16 h post-inoculation and the expression of CD40, CD86, CD83 and CD80 were analyzed by flow cytometry. Isotype-matched controls were shown.

**Figure 5:**
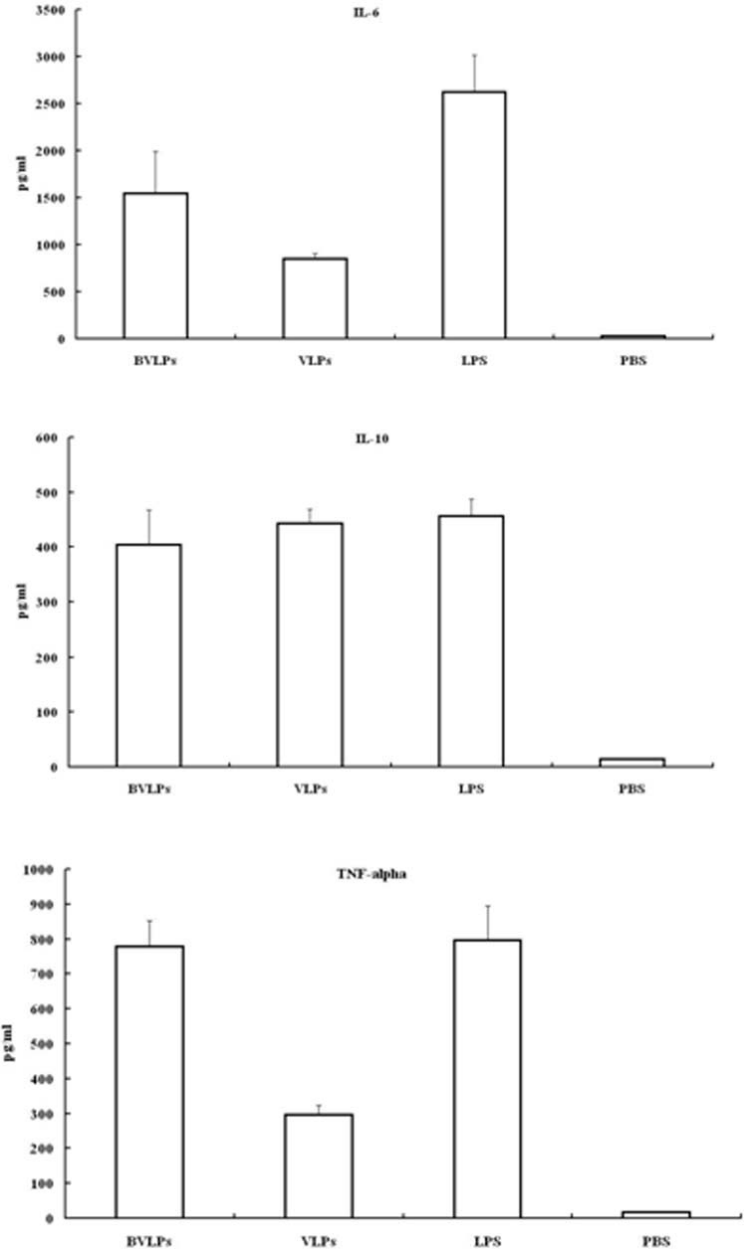
Cytokines secretions in DCs treated with BVLPs or with SARS CoV VLPs. DCs were incubated with SL-CoV BVLPs or SARS CoV VLPs at a concentration of 10 µg/ml. DCs treated with LPS (10 µg/ml) and PBS were used as positive and negative control, respectively. After 16 h incubation, supernatants were collected, and IL-6, IL10 and TNF-α were analyzed using ELISA. Data are expressed as the mean±SD of triplicate samples.

We previously constructed SARS CoV VLPs and investigated the humoral and cellular immune responses induced by SARS CoV VLPs in mice [29]. Whether SARS CoV VLPs can activate DCs remains to be addressed. In the current study, 10 µg/ml of either SARS CoV VLPs or BVLPs was incubated with immature DCs, and the expression of surface makers and the secretion of cytokines were subsequently analyzed. Similar to LPS treatment, both SARS CoV VLPs and BVLPs enhanced the expression of CD40, CD86, CD80 and CD83 (Fig. 4). However, BVLPs showed a stronger ability to induce the secretion of IL-6 and TNF-α, with 2 and 4 fold higher, respectively, while the secretion of IL 10 was similar (Fig. 5). Because the only difference between SARS CoV VLPs and BVLPs lies in the S protein, the difference in IL-6 and TNF-α induction is highly likely due to the variation between the two S proteins.

### TH1/TH2 differentiation of T cells stimulated by DCs treated with BVLPs or SARS CoV VLPs

**Figure 6:**
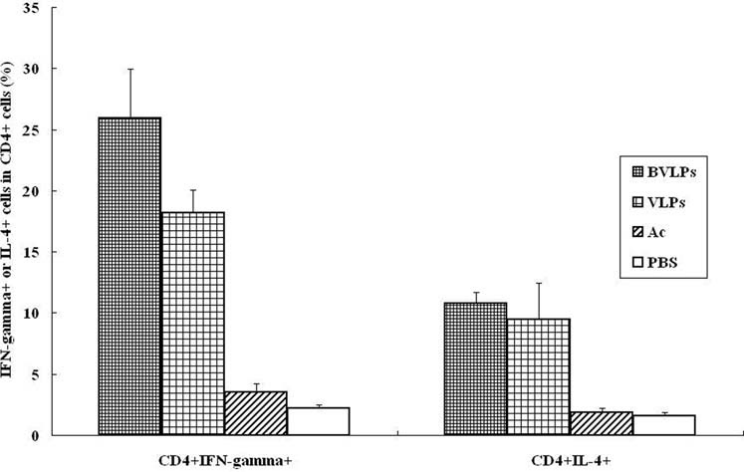
Evaluation of CD4 T cell types stimulated by BVLPs-exposed DCs. Immature DCs (1×10^4^) were exposed to BVLPs (10 µg/ml), Ac or PBS for 24 h and then co-cultured with CD4+T cells (1×10^5^) in 96-well plates in triplicate for another 72 h. The DCs/T mixtures were then collected for intracellular IFN-γ or IL-4 staining followed by flow cytometry analysis. IFN-γ and IL-4 positive populations in CD4 T cells were analyzed. Data are expressed as the mean±SD of triplicate samples.

To understand the types of T cells stimulated by VLPs-exposed DCs, IFN-γ and IL-2 intracellular staining was measured by flow cytometry. As show in Fig. 6, IFN-γ+ population in CD4+ T cells stimulated by either BVLPs- or SARS CoV VLPs-treated DCs were much higher than IL-4+ popoulation. In PBS- or Ac-treated groups, the percentage between IFN-γ+ and IL-4+ populations in CD4+ T cells did not have significant difference. Thus, the BVLPs or SARS CoV VLPs treated DCs could polarize Th cells toward a Th1 phenotype. The balance between Th1 and Th2 responses to an infectious agent can influence both pathogen growth and immunopathology. Th1-dominant cellular immunity may be a crucial factor for long-term protection against virus infection.

### S-specific antibody and Th1/Th2 type responses

**Figure 7:**
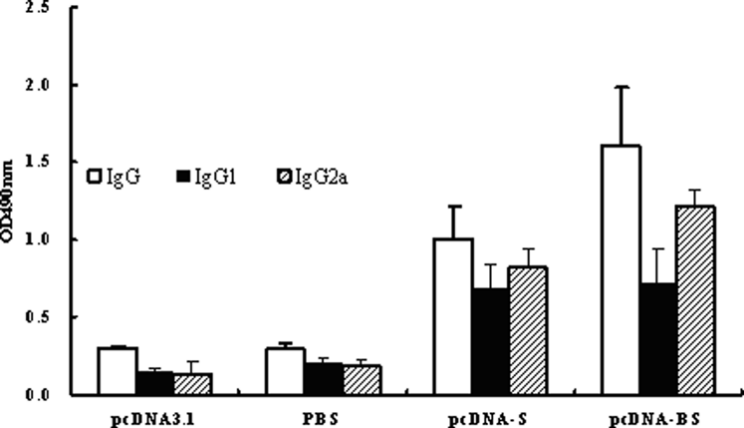
Detection of SARS-CoV S-specific IgG and the subclasses in vaccinated mice. Mouse sera (8 per group) were collected 10 days after the final immunization and assayed for IgG1 and IgG2a against the S2 protein of SARS-CoV. ELISA was used to detect the level of S-specific IgG antibodies. Recombinant S2 protein expressed in *E. Coli* was purified and used as the detection antigen. Data are presented as means±SD.

The observed difference in DC-stimulating activity between SARS CoV VLPs and BVLPs was very likely due to the S protein. To further confirm our conclusion, we cloned *s* and *bs* gene into pcDNA3.1 to construct the recombinant plasmids pcDNA-S and pcDNA-BS, respectively. These two plasmids were then immunized in mice intramuscularly every two weeks. Ten days after the final immunization, IgG1 and IgG2a were measured to determine the humoral immune response profiles by ELISA. As shown in Fig. 7, the anti-SARS-CoV IgG subtype profile revealed that both IgG1 and IgG2a were induced in all the immunization regimens. Although the level of IgG1 was similar, the level of IgG2a in the pcDNA-BS vaccinated group was appreciably higher than that in pcDNA-S immunized group.

**Figure 8:**
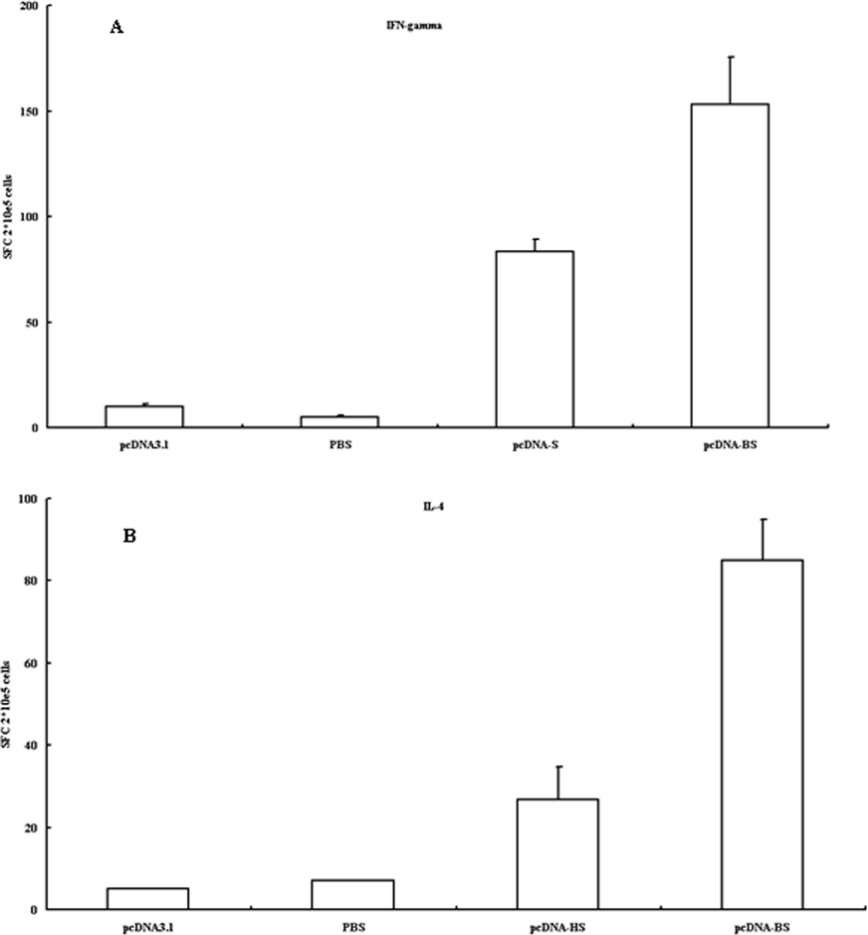
SARS-CoV S protein-specific IFN-γ and IL-4 ELISPOT. The number of INF-γ or IL-4 -secreting cells was assayed by ELISPOT using splenocytes harvested from mouse spleens 10 days after the final immunization and stimulated *in vitro* with purified S2 protein. The results represent the averages of triplicate wells of 3 mice and are expressed as means±SD. (A) SARS-CoV S protein-specific IFN-γ-secreting cells. (B) SARS-CoV S protein-specific IL-4-secreting cells.

ELISPOT assay was also used to assess the magnitudes of S-specific IFN-γ (Th1) and IL-4 (Th2) T-cell responses after the mice were vaccinated with pcDNA-S or pcDNA-BS. Compared with pcDNA-S immunization, antigen-specific IFN-γ-secreting cell number was 2-fold higher and IL-4-secreting cell number was 3 times higher in mice immunized with pcDNA-BS (P<0.05) (Fig. 8). Moreover, in all groups, IFN-γ was induced to a much higher level than that of IL-4. Combing with the higher IgG2a elevated by BS immunization, it is reasonable to draw a conclusion that BS protein could induce a stronger Th1 bias immune response than S protein.

## Discussion

The spike (S) protein of SARS CoV is a key protein involved in viral entry and therefore the main target for vaccine design [32] http://www.ncbi.nlm.nih.gov/entrez/query.fcgidbpubmedcmdSearchitoolpubmed_AbstractPlusterm22ZhaoP225BAuthor5D. In the absence of S protein, expression of other structure proteins, such as M and E proteins, does not induce a detectable neutralizing antibody response [33]. Antibodies against SARS CoV S protein inhibit pulmonary viral replication and protect against SARS CoV challenge [34]. Variations in the S proteins can dramatically affect the virulence and host tropism of the virus [35], [36]. For instance, SL-CoVs isolated from bats display greater genetic variations than SARS CoVs from humans or civets [19]. Here, we intended to investigate whether the S protein of SL-CoV isolated from bats can incorporate into viral like particles (VLPs) formed by the E and M proteins of human SARS CoV and whether such VLPs can activate DCs.

Using baculovirus expression system, we constructed BVLPs incorporated with the E and M proteins of human SARS CoV and the S protein of SL-CoV of bats. Electron microscopy (EM) detection confirmed the formation of VLPs, which were very similar to the morphology of the native virus. Western blot indicated that BS protein was incorporated into BVLPs. Because the major difference of S proteins between SARS CoV and SL-CoV locates in the S1 region (∼64%) and the S2 region is more conserved (92% to 96%) [19], [20], successful incorporation of the SL-CoV S protein into VLPs formed by the E and M proteins of SARS CoV suggests that such BVLPs likely assembled through S2 region other than S1 region. These genome-free BVLPs formed by structure proteins from different species provide us a physical means to investigate their immunogenicity in immune cells.

SARS CoV mainly targets lung tissues and the clinical syndrome of SARS indicates that the host immune system is greatly damaged [37]–[39]. Dendritic cells (DCs) are antigen-presenting cells that play key role in innate and adaptive immunity [31], [40]–[42]. Exposure of DCs to infectious SARS CoV leads to the phenotypic and functional maturation of DCs, including costimulatory molecule expression, T cell-stimulation and cytokine production. [43]. In addition, UV-inactivated SARS CoV also activates immature DCs [44]. In this study, we have demonstrated that BVLPs, morphologically and antigenically similar to the native virus, exhibited stimulating ability in DCs and induced DC maturation by enhancing the expression of cell-surface costimulatory molecules including those essential for optimal activation of T cells: CD40, CD86, CD80 and CD83. Phenotypic maturation of DCs exposed to BVLPs was similar to those observed in DCs incubated with lipopolysaccharide (LPS), a well-known stimulator of DCs maturation. In contrast, heat-denatured BVLPs lost the ability to enhance the expression of cell surface molecules and the secretion of cytokine, suggesting that the specific structure of viral proteins might significantly contribute to the immune modulating activity. Taken together, our data indicate that the baculovirus derived-BVLPs can interact with human DCs to induce DC maturation and activation.

Our previous study found that VLPs formed by the S, E, and M proteins of human SARS CoV elicited strong SARS CoV-specific humoral and cellular immune responses in mice and conferred protective immunity against the infection of S protein -pseudotyped murine leukemia viruses (MLV) [29]. Here, we showed that both BVLPs and SARS CoV VLPs activated DCs and induced DC maturation by enhancing the expression of costimulatory molecules (CD40, CD86 and CD80) and the maturation marker CD83. A study in SARS CoV-infected patients indicated that the levels of IL-6 and TNF-α were markedly increased in the acute stage and returned to normal after given adequate immunosuppressive treatment [45]. Corticosteroid has been used for the treatment of SARS on the basis that proinflammatory cytokines is responsible for the immunopathology in the lungs [46]. Of interest, in the current study, BVLPs induced much higher levels of IL-6 and TNF-α than did SARS CoV VLPs. It seemed that BVLPs vs DCs served as a better model to simulate the acute-phase response between the virus and the host. Thus, this model may be applicable in the development of vaccines and drugs against the virus. TNF-α and IL-6 are important proinflammatory cytokines mediating apoptosis, immunity, and inflammation, and may play important role in antiviral responses and inflammation [31]. A previous study by Wang et al indicated that the S protein of SARS CoV played a key role in the production of proinflammatory cytokine at a stage of virus-host cell interaction. S protein could activate NF-κB signaling pathway and thus up-regulate the secretion of IL-6 and TNF-α. Here we demonstrate that BVLPs are stronger immune modulators in terms of DC activation and maturation. Since the only difference between SARS CoV VLPs and BVLPs was the S protein incorporated, the enhanced immune modulating ability of BVLPs is very likely contributed by the S protein.

Different types of DCs may play different roles in the onset of a Th1 or Th2 immune response. The balance between Th1 and Th2 cells is important and may determine the outcome of an immune response toward infection and allergy. We therefore studied the influence of BVLPs-stimulated DCs on T cell polarization by intracellular cytokine staining. Compared to mock-treated DCs, BVLPs-exposed DCs increased the population of IFN-γ or IL-4 positive CD4 T cells. The IFN-γ secretion level was much higher than that of IL-4, indicating a bias of Th1 immune response. Because matured DCs can enhance native T cells differentiation, these results further confirm that BVLPs can induce DCs activation and maturation.

BVLPs had stronger ability to stimulate DCs by inducing the production of IL-6 and TNF-α in our study. To address whether such difference was S protein specific, we constructed two recombinant plasmids pcDNA-S and pcDNA-BS, and detected their immune responses in mice. The higher level of IgG2a and the higher number of S protein-specific IFN-γ or IL-4-secreting cells in mice immunized with pcDNA-BS indicated that the S protein of bat SL CoV was more immunogenic. Our in vivo data provide evidence that the difference of immune modulating ability between BVLPs and SARS-CoV VLPs was highly likely contributed by the S protein.

Taken together, we have successfully constructed BVLPs formed by membrane proteins of different origins, one from SL-CoV isolated from bats (BS) and the other two (E and M) from human SARS CoV. The formed BVLPs demonstrated stimulating activity in immature DCs. In the absence of an in vitro cell culture model for SL-CoV, our study has together demonstrated that BVLPs vs DCs can be used as a model to study the interaction between SL-CoV and host cells. Further study by using this model may provide important information for vaccine development and for understanding the pathogenesis and the evolution of SL-CoV.

## Materials and Methods

### Construction of plasmids and recombinant baculoviruses

DNA coding the S protein (BS) of Bat SL-CoV was amplified from Rp3 strain (GenBank accession number DQ071615) by reverse transcription-polymerase chain reaction (RT-PCR) using the following primers: 5′-TTTGGATCCCCACCATGAAAATTTTAATTCTT and 5′-GGG GAATTCTTATGTGTAGTGTAATTTTAC. *bs* gene was cloned into pFastBac DUAL vector [47] under the control of a strong viral promoter, polyhedron promoter. *gfp* gene was cloned under the control of p10 promoter to generate pFBS. Recombinant baculoviruses were generated using Autographa californica multiple nucleopolyhedrovirus (AcMNPV) genome. In brief, vAcBS was generated by transfecting the BS plasmids into insect cells sf21 using Lipofectin (Invitrogen). Recombinant baculovirus vAcME was generated as previously described [30].

In addition, *s* and *bs* gene were subcloned into pcDNA3.1(+) (Invitrogen, Carlsbad, Calif.) to construct the recombinant plasmids pcDNA-S and pcDNA-BS, respectively. The accuracy of the constructs was confirmed by restriction digestion and sequencing. DNA plasmids were purified using Qiagen MegaPrep columns (Qiagen) and dissolved in endotoxin-free PBS to a final concentration of 2 µg/µl and stored at −20°C.

### Generation of SARS-CoV like particles containing BS (BVLPs)

Insect cells were co-infected with vAcBS and vAcME at a multiplicity of infection (moi) of 5. At 4 days post-infection, cells and medium were harvested and centrifuged at 90,100 g for 4 hours. BVLPs in the pellets were resuspended in PBS and further purified on a discontinuous 30–50% (w/v) sucrose gradient. A visible band between 30% and 40% sucrose layers was collected, concentrated by centrifugation and then resuspended in PBS at a total protein concentration of 2 µg/µl (measured in Eppendorf Biophotometer, Germany). The purity of the VLPs was then checked by SDS-PAGE. The presence of BS proteins in the purified preparations was confirmed by western blot using rabbit-derived anti-SARS-CoV antibody, kindly provided by Prof. Lin-Fa Wang in Australian Animal Health Laboratory (B.T. Eaton and L.-F. Wang, unpublished results). Immunogold labeling was detected using antiserum against BS2 (667–1242 aa in S2 region of Bat S protein). To generate BS2 antiserum, the cDNA of 667–1242 aa in S2 region was cloned into pMAL-c2x expression vector (NEB). BS2 protein was purified by using Amylose Resins (NEB) and then was used to immunize rabbit.

### Characterization of BVLPs by electron microscopy (EM)

For EM study of BVLPs budding from insect cells, Sf21 cells were co-infected with vAcBS and vAcME at a MOI of 5. At 72 h post-infection, the cells were collected and fixed with 2% glutaraldehyde and then with 1% osmium tetroxide. Thin-section samples were prepared and examined under a Hitachi-H7500 transmission electron microscope. For immunogold labeling, BVLPs were loaded onto a collodion-coated EM grid for 5 min. After removal of the excess sample solution, antibody against BS2 protein was added onto the grid and incubated for 1 h at room temperature. Grids were washed three times and then incubated with 15 nm gold conjugated anti-rabbit IgG for 1 h. The samples were stained with 2% phosphotungstic acid (PTA) for 1 min, then drained, and examined under the EM.

### Culture of primary human dendritic cells (DCs)

Human peripheral blood mononuclear cells (PBMC) were isolated from healthy donors by Isopaque-Ficoll (Lymphoprep; Nycomed, Oslo, Norway) following the manufacturer's instructions. Immature DCs were generated from PBMC as previously described [48] with minor modifications. Briefly, PBMC in RPMI 1640 (Gibco Laboratories, Grand Island, NY) were cultured in six-well plates (2×10^6^ cells/ml) for 2 hours. Adherent monocytes were washed with RPMI 1640 and then cultured for 6 days in complete medium containing 10% fetal calf serum (Life Technologies) and supplemented with rhGM-CSF (1000 U/ml) and rhIL-4 (500 U/ml) (both from Peprotech). Half of the medium was replaced every two days. The resulting differentiated DCs were >97% CD1a positive and <1% CD14 positive.

### Ethics

All participants gave written informed consent. This study was conducted in accordance with the Declaration of Hubei and approved by Health Department of Hubei Province and Wuhan Blood Center of Hubei Province, China.

### Stimulation of DCs with BVLPs

DCs (2×10^6^ cells) were incubated with either 10 µg/ml of BVLPs or 10 µg/ml of lipopolysaccharide (LPS, Sigma, St. Louis, MO). Meanwhile, immature DCs were treated with PBS, Ac (culture supernatant of sf21 cells transfected with baculovirus expression vector pFastBac DUAL with *gfp* under the control of p10 promoter) or heat-denatured BVLPs (100°C for 10 min). Alternatively, an equivalent amount of BVLPs or LPS was incubated with 10 µg/ml polymyxin B (a compound that binds to LPS and inactivates LPS's biological functions [49]) at room temperature for 1 h before added to DCs. After different treatments of DCs at 37°C for 16 h, supernatants were removed and frozen at −70°C until cytokine detection. DCs were stained for specific cell-surface markers and analyzed by flow cytometry.

### Assay of cytokines

Culture supernatants were collected for cytokine (TNF-a, IL-6 and IL-10) quantification using enzyme-linked immunosorbent assay (ELISA) according to the manufacturer's instructions (U-CyTech, sensitivity: 2 pg/ml).

### Flow cytometry

DC surface markers were analyzed by flow cytometry. Briefly, cells were washed in cold PBS and cell surface staining was carried out using the following antibodies: fluorescein isothiocyanate (FITC)-conjugated anti-CD40, FITC-conjugated anti-CD80, phycoerythrin (PE)-conjugated anti-CD83, PE-conjugated anti-CD86 (all from PharMingen). After incubation with antibodies for 30 min at 4°C, cells were washed and analyzed using flow cytometer (Beckman, EPICS ALTRA, USA). Data were analyzed using EXOPO analysis software. A minimum of 10,000 events were collected and analyzed for each sample. Isotype-matched controls (PE-conjugated mouse-anti-human-IgG1, FITC-conjugated mouse-anti-human-IgG1, both from BD PharMingen) were included in each staining.

### Evaluation of T cell types stimulated by BVLPs-exposed DCs

CD4+T cells were prepared from PBMC by positive selection using anti-CD4 antibody (BD PharMingen) and the purity was consistently above 95%. Immature DCs (1×10^4^) were exposed to BVLPs (10 µg/ml), Ac or PBS for 24 h and washed thoroughly. Treated DCs were then co-cultured with CD4+T cells (1×10^5^) in 96-well plates (Costar) in triplicate for another 72 h. For intracellular detection of IL-4 and IFN-γ, DCs/T cell mixtures were further stimulated with 10 ng/mL PMA (phorbol 12-myristate 13-acetate) and 0.5 µmol/L ionomycin for 6 h. After stimulation, mononsesin (1.5 µl/ml) was added to each well and plates were incubated at 37°C for 3 h to block reaction. The cells were then washed, fixed and permeabilized. Intracellular IFN-γ and IL-2 (both PE-conjugated) staining and flow cytometry analysis (Beckman, EPICS ALTRA, USA) were performed in succession. Data were analyzed using EXOPO analysis software. A minimum of 10,000 events were collected and analyzed for each sample. Isotype-matched controls (PE-conjugated mouse-anti-human-IgG1, BD PharMingen) were included in each staining.

### Analysis of the humoral immune response

6–8 wk-old female BALB/c mice were immunized intramuscularly with purified plasmid pcDNA-S or pcDNA-BS 3 times at a 2-wk interval. S-specific IgG and the subclasses (IgG1 and IgG2a) in serum were assessed by ELISA. Recombinant S2 protein expressed in *E. Coli* was purified and used as the detection antigen. Horseradish peroxidase (HRP)-conjugated goat anti-mouse IgG, IgG1 or IgG2a (sigma) was used as the secondary antibody. Optical density (OD) was read at 490 nm (A_490_).

### SARS-CoV S-specific ELISPOT assay

Cellular immune responses to SARS-CoV were assessed by IFN-γ and IL-4 ELISPOT assays using mouse splenocytes. Assays were performed according to the instruction manual (U-CyTech, Netherlands). Absorbance was read using an ELISPOT reader (Hitech Instruments) and spot-forming cells (SFC) per 10^6^ splenocytes were calculated. The medium background response in the absence of antigen was consistently <10 SFC per 10^6^ splenocytes.

### Statistical analysis

All data were presented as means±SD for the immunized mice per group. The SPSS 13.0 software for windows was used for statistical analysis. The LSD t-test was used for between group comparisons. P values<0.05 were considered statistically significant.
